# Linear Solvation Energy Relationships in the Determination of Specificity and Selectivity of Stationary Phases

**DOI:** 10.1007/s10337-012-2310-9

**Published:** 2012-09-15

**Authors:** S. Studzińska, B. Buszewski

**Affiliations:** Chair of Environmental Chemistry and Bioanalytics, Faculty of Chemistry, Nicolaus Copernicus University, 7 Gagarin St., 87-100 Toruń, Poland

**Keywords:** High performance liquid chromatography, Linear solvation energy relationships, Specific stationary phase, Cavity factor, Hydrogen bond acceptor basicity

## Abstract

The retention of fifty structurally different compounds has been studied using linear solvation energy relationships. Investigations were performed with the use of six various stationary phases with two mobile phases (50/50 % v/v methanol/water and 50/50 % v/v acetonitrile/water). Packing materials were home-made and functionalized with octadecyl, alkylamide, cholesterol, alkyl-phosphate and phenyl molecules. This is the first attempt to compare all of these stationary phases synthesized on the same silica gel batch. Therefore, all of them may be compared in more complex and believable way, than it was performed earlier in former investigations. The phase properties (based on Abraham model) were used to the classification of stationary phases according to their interaction properties. The hydrophilic system properties *s*, *a*, *b* indicate stronger interactions between solute and mobile phase for most of the columns. Both *e* and *v* cause greater retention as a consequence of preferable interactions with stationary phase by electron pairs and cavity formation as well as hydrophobic bonds. However, alkyl-phosphate phase has different retention properties, as it was expressed by positive sign of *s* coefficient. It may be concluded that most important parameters influencing the retention of compounds are volume and hydrogen bond acceptor basicity. The LSER coefficients showed also the dependency on the type of organic modifier used as a mobile phase component.

## Introduction

Chromatographers working with high performance liquid chromatography (HPLC) look for a better understanding of various interactions taking place during the chromatographic process. Several different methods like functional group contributions [1], principal component analysis [2] and chemometric methods [1, 3] have been used for such purposes. However, probably the most popular method applied in HPLC is the solvation parameter model by Abraham [4]. It is based on the linear solvation energy relationships (LSERs) and allows obtain information about the stationary phase retention properties. The solvation parameter model may be described by equation:

1 $$ \log {\text{SP}} = c + eE + sS + aA + bB + vV $$

where: log SP is the property of a series of analytes, *E* is an excess molar refraction, *S* the solute dipolarity/polarizability, *A, B* the overall or effective hydrogen-bond acidity and basicity, *V*—the McGowan characteristic volume.

Coefficients *e, s, a, b, v* in Abraham equation are derived by multiple linear regression (MLR) analysis. These constants reflect the solvation properties and consequently: *e* is the ability of the solvent to interact with electron pairs, *s* is the solvent dipolarity/polarizability, *a* is the solvent hydrogen-bond basicity, *b* is the solvent hydrogen-bond acidity, *v* refers to the ability to interact with a methylene group, consequently *v* is a measure of solvent lipophilicity [5]. These are five interactions causing differences in retention mechanism and cause suitable or not suitable selectivity and resolution.

Constants in Eq. (1) represent specific interactions between analyzed solute and mobile or stationary phase: electron pair (*e*), dipole or dipole-induced-dipole and polarizability interaction (*s*), solute acid-solvent base (*a*) and solute base-solvent acid (*b*), differences in cavity effects and hydrophobic interactions (*v*) [6]. Most of the descriptors may be obtained experimentally from gas–liquid chromatographic (GLC) data for solutes and from water-solvent partition coefficients for solutes in general [1–4, 6]. Other descriptors can be simply calculated for analyte on the basis of its molecular structure [6].

Linear solvation energy relationship has been used for many analytical purposes; however, the main advantage is the study of the chromatographic system characterization [7–13]. It was also applied for the investigation of retention behavior of drugs and many other biologically important compounds [14–17]. LSER model is therefore very useful when new stationary phases are tested for the analysis of various substances.

The significant development in the synthesis of new, specific packings for HPLC has been observed during the last decades. It concerns silica-based, polymeric, chiral, zwitterionic and biological membrane imitating materials [18–21]. It is connected with various interactions taking place during the chromatographic processes e.g. ion–ion, ion–dipole, dipole–dipole, hydrogen bonding, electron pair donor-electron pair acceptor. Determination of which one is the predominant is very difficult and detailed studies are necessary.

For this reason the main aim of present study was the investigation of retention of 50 analytes with the use of Abraham model on six different HPLC packing materials. They have been specially synthesized on the same batch of silica gel for the purposes of present investigation. Received packings are functionalized with octadecyl chains (of two densities), alkylamide, cholesterol, alkyl-phosphate, phenyl groups. Although most of these stationary phases are well known, they were never compared in one study. Only commercially available phases were studied. Application of LSER allows to establish which type of interactions will be mainly responsible for the retention of analytes. The properties of specific phases were compared with nonpolar octadecyl packings. Acetonitrile and methanol were used as mobile phase components to compare the influence of both organic solvents on interactions occurring between solute, stationary and mobile phase.

## Materials and Methods

### Materials

A set of 50 compounds was used in the experiments. Test solutes were taken from several sources: Sigma-Aldrich (Gillingham, Dorset, UK), Merck (Merck, Darmstadt, Germany) and from the collection of Chair of Organic Chemistry, Faculty of Chemistry, Nicolaus Copernicus University. Their names and descriptors are listed in Table 1. The *E*, *S*, *A*, *B*, *V* values were taken from literature [6, 22, 23]. The stock solutions of standards were prepared by dissolving a weighed amount in methanol. Concentrations of analytes used for retention studies were in the range of 10–40 μg ml^−1^. Most of the solutes were detected at a wavelength of 254 nm and some of them at 210 nm.

**Table 1:** Test solutes and their parameters taken from [6, 20, 21]

Solute	*R*	π_2_^*H*^	α_2_^*H*^	β_2_^*H*^	*V*_*x*_
*n*-Butyl acetate	0.071	0.60	0	0.45	1.0284
*n*-Pentyl acetate	0.067	0.58	0	0.45	1.1693
2-Propanone	0.179	0.70	0.04	0.49	0.547
Butan-2-one	0.166	0.70	0	0.51	0.6879
Hexan-2-one	0.136	0.68	0	0.51	0.9697
Heptan-2-one	0.123	0.66	0	0.51	1.1106
Chloroform	0.425	0.49	0.15	0.02	0.6167
Phenylmethanol	0.803	0.87	0.39	0.56	0.916
Benzaldehyde	0.820	1.00	0	0.39	0.873
Methyl benzoate	0.733	0.85	0	0.46	1.0726
Methoxybenzene	0.708	0.75	0	0.29	0.916
1-Phenylethanone	0.818	1.01	0	0.48	1.0139
1-Phenylpropan-1-one	0.804	0.95	0	0.51	1.1548
Diphenylmethanone	1.447	1.50	0	0.50	1.4808
2-Phenylacetonitrile	0.751	1.15	0	0.45	1.012
Nitrobenzene	0.871	1.11	0	0.28	0.8906
*p*-Nitrotoluene	0.87	1.11	0	0.27	1.0315
Fluorobenzene	0.477	0.57	0	0.10	0.7341
Chlorobenzene	0.718	0.65	0	0.07	0.8388
Bromobenzene	0.882	0.73	0	0.09	0.9814
Iodobenzene	1.188	0.82	0	0.12	0.9746
1-Chloro-4-methylbenzene	0.705	0.67	0	0.07	0.9797
Benzene	0.610	0.52	0	0.14	0.7164
Methylbenzene	0.601	0.52	0	0.14	0.8573
Ethylbenzene	0.613	0.51	0	0.15	0.9982
Propylbenzene	0.604	0.50	0	0.15	1.1391
Butylbenzene	0.600	0.51	0	0.15	1.280
1,4-Dimethylbenzene	0.613	0.52	0	0.16	0.9982
Biphenyl	1.360	0.99	0	0.26	1.3242
1,3,5-Trimethylbenzene	0.649	0.52	0	0.19	1.1391
Bicyclo[4.4.0]deca-1,3,5,7,9-pentene	1.340	0.92	0	0.20	1.0854
Phenol	0.805	0.89	0.60	0.30	0.7751
3-Methylphenol	0.822	0.88	0.57	0.34	0.916
4-Methylphenol	0.82	0.87	0.57	0.31	0.916
2-Methylphenol	0.84	0.86	0.52	0.30	0.916
4-Ethylphenol	0.800	0.90	0.55	0.36	1.0569
4-Chlorophenol	0.915	1.08	0.67	0.2	0.8975
2-Chlorophenol	0.853	0.88	0.32	0.31	0.8975
3-Chlorophenol	0.909	1.06	0.69	0.15	0.8975
3,5-Dichlorophenol	1.020	1.10	0.83	0	1.02
4-Iodophenol	1.380	1.22	0.68	0.20	1.033
Phenylamine	0.955	0.96	0.26	0.41	0.8162
*N,N*-Dimethylaniline	0.957	0.81	0	0.41	1.098
1,2-Dimethylbenzene	0.663	0.56	0	0.16	0.998
Benzamide	0.990	1.50	0.49	0.67	0.9728
Benzonitrile	0.742	1.11	0	0.33	0.8711
2-Methylaniline	0.966	0.92	0.23	0.45	0.957
3-Methylaniline	0.946	0.95	0.23	0.45	0.957
4-Methylaniline	0.923	0.95	0.23	0.45	0.957
Furan	0.369	0.53	0	0.13	0.5363

The mobile phases were prepared of methanol and acetonitrile of gradient grade purity (J. T. Baker, Deventer, Holland) and deionized water from Milli-Q system (Millipore, El Passo, TX, USA).

### Apparatus and Analysis Conditions

The UltiMate^®^ 3000 Binary Rapid Separation LC (RSLC) (Dionex, Sunnyvale, CA, USA) ultra high performance liquid chromatography system equipped with a diode-array detector was chosen for chromatographic measurements. Chromeleon 7 program was used for the data collection.

Chromatographic analysis was carried out with isocratic conditions for two different mobile phase compositions. The first mobile phase (MP1) consisted of methanol and water 50/50 % v/v, while the second one (MP2) was a mixture of acetonitrile and water 50/50 % v/v. The flow rate was 1 ml min^−1^. The void volume of the system was determined with the injection of uracil or thiourea. The temperature of autosampler and column was 20 °C.

### Stationary Phases

**Fig. 1:**
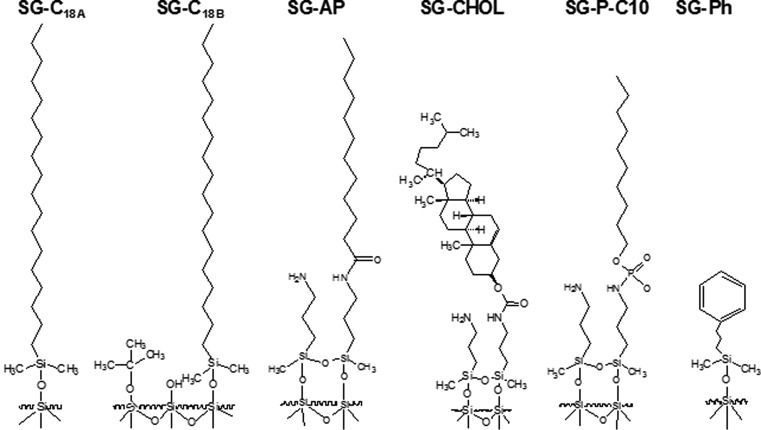
Schematic structures of stationary phases used in the study

In the current study six HPLC columns have been used: two octadecyl ones, alkylamide, cholesterolic, alkyl-phosphate, phenyl. Their detailed characteristics is presented in Table 2. All stationary phases were prepared in our laboratory. The synthesis was performed according to the reaction mechanism and the conditions described earlier: octadecyl in [24], alkylamide [25], cholesterolic [26], alkyl-phosphate [27], phenyl [28]. Figure 1 presents structures of chemically bonded stationary phases. They were prepared on the basis of the same batch of silica gel Kromasil^®^. Its physicochemical characteristic was published earlier in [24–27]. The received packing materials were packed into 125 × 4.6 mm I.D. stainless-steel tubes using home-made apparatus equipped with Haskel packing pump (Burbank, CA, USA) under constant pressure.

**Table 2:** Detailed characteristics of stationary phases used in the investigations

Type of stationary phase	Column	Shortcut	Column dimensions (mm)	Silica particle size (μm)	Pore diameter (Å)	Proportional part			
						I modification step		II modification step	
						Carbon P_C_ (%)	Nitrogen P_N_ (%)	Carbon P_C_ (%)	Nitrogen P_N_ (%)
Octadecyl	Octadecyl	SG-C_18A_	125 × 4.6	5	100	17.79	–	–	–
Octadecyl	Octadecyl, end-capped	SG-_18B_	125 × 4.6	5	100	7.55	–	–	–
Alkylamide	Alkylamide	SG-AP	125 × 4.6	5	100	4.47	6.65	11.46	–
Cholesterol	Cholesterolic	SG-CHOL	125 × 4.6	5	100	4.47	6.65	17.82	4.47
Alkyl-phosphate	Alkyl-phosphate	SG-P-C10	125 × 4.6	5	100	2.83	1.25	8.43	0.91
Phenyl	Phenyl	SG-Ph	125 × 4.6	5	100	11.75	–	–	–

### Multiple Linear Regression Analysis

The multiple regression procedure has been performed using the Statistica 8.0 package (StatSoft, Tulsa, USA). Multiple regression was chosen and results presented in the paper concern the best subset model building.

## Results and Discussion

### Test Solute Selection

A large number of various compounds were chosen for LSER studies. The main criteria of selection was to collect both aromatic and aliphatic substances with a wide range of properties. Chosen compounds differ in size, dipolarity/polarizability, hydrogen bond donor or acceptor characteristics as it is presented in Table 1. Such collection is of great importance, especially when the significance of LSER equations is considered. These compounds were chosen to avoid several effects commonly observed for Abraham model, e.g., high correlation of polarity with the solute size (therefore a high percentage of low polarity compounds was not selected).

To avoid problems with the variance during the multiple regression analysis, the analyte parameters (*E, S, A, B, V*) cannot covary. Therefore, we have appointed the variance–covariance matrix, which is presented in Table 3. It can be seen that solute descriptors are weakly correlated. Because of low covariances it may be concluded that the data sets are free of statistically significant artifacts.

**Table 3:** Correlation coefficient matrix of solute variables used in LSER equations

	*R*	π_2_^*H*^	α_2_^*H*^	β_2_^*H*^	*V*_*x*_
*R*	1.00	0.63	0.22	−0.21	0.35
π_2_^*H*^	0.63	1.00	0.38	0.33	0.13
α_2_^*H*^	0.22	0.38	1.00	−0.05	−0.11
β_2_^*H*^	−0.21	0.33	−0.05	1.00	0.11
*V*_*x*_	0.35	0.13	−0.11	0.11	1.00

### Stationary Phases Selection

We have prepared home-made stationary phases. Therefore, we were able to characterize them with the use of several spectroscopic techniques and elemental analysis. A number of information concerning the structure and percentage part of carbon and nitrogen content was collected (Table 2). Next these supports were packed into similar tubes. The rare collection of various columns synthesized in one laboratory with the same silica gel was obtained. Therefore, the comparison of different home-made stationary phases from the point of view of interactions by the means of LSER could be performed. Most of the results of LSER presented in the literature concern just a comparison of commercially available stationary phases. Such comparison is not complex, especially when HPLC columns were purchased from various manufacturers.

We have used two different octadecyl phases (Fig. 1; Table 2). Both of them have different carbon load. SG-C_18B_ has about 10 % more carbon in comparison with SG-C_18A_. In the same time SG-C_18B_ has similar carbon content as SG-CHOL. SG-C_18A_ and SG-C_18B_ contain on silica surface long alkyl chain and residual silanols. More complicated structure is typical for other stationary phases. SG-AP, SG-CHOL, and SG-P-C10 were synthesized in two steps, first one was similar for all of them and consisted of bond creation between silanols and aminopropyl groups. Next SG-AP, SG-CHOL, and SG-P-C10 were synthesized depending on what kind of functional group was bonded to aminopropyl surface (Fig. 1). Therefore, these three columns are somewhat similar in structure; however, alkylamide, cholesterol or phospho-alkyl group has significant and various effect on retention. Phenyl stationary phase SG-Ph was also synthesized in our laboratory and used to compare the influence of π–π interaction, as it was interesting to test also this type of packing.

The collection of stationary phases synthesized with the use of the same silica gel gives the ability to more complex and appropriate comparison of received materials with each other. Octadecyl, cholesterol and phenyl stationary phases were already tested with the use of LSER model; however, those attempts were performed with commercial phases. We present for the first time results obtained for group of home-made stationary phases. On the other hand alkyl-phosphate packing was studied with the use of Abraham equation for the first time.

### Test Solutes Retention

Table 4 collects all log *k* values for columns and two mobile phases (MP1 and MP2) used in the study. Typical tendency concerning higher retention of analytes for mobile phase containing methanol was observed. Moreover, most of the compounds were retained with the greatest extent inside SG-C_18B_ column. The retention strength for most of the compounds decreased in the order of SG-CHOL > SG-AP > SG-Ph > SG-C_18A_ > IAM > SG-P-C10. However, there were some analytes, which had the highest log *k* values on SG-Ph (e.g. aryl ketones) or SG-CHOL (chlorophenols, iodophenols, aniline, nitrotoluene, nitrobenzene). Surprisingly, the lowest retention of most of the solutes was achieved for SG-P-C10, although it does not contain the lowest carbon load among all the packings used in the study.

**Table 4:** Log *k* values In MP1 and MP2 for all columns used in the investigations

Solute	log *k*											
	SG-C_18A_		SG-C_18B_		SG-AP		SG-CHOL		SG-P-C10		SG-Ph	
	MP1	MP2	MP1	MP2	MP1	MP2	MP1	MP2	MP1	MP2	MP1	MP2
*n*-Butyl acetate	0.273	−0.002	0.847	0.468	0.302	0.170	0.572	0.233	−0.533	−0.897	0.509	0.210
*n*-Pentyl acetate	0.531	0.169	1.157	0.680	0.533	0.318	0.828	0.404	−0.329	−0.819	0.745	0.310
2-Propanone	−0.915	−0.791	−0.745	−0.573	−0.670	−0.396	−0.709	−0.561	−1.210	−1.100	−0.486	−0.528
Butan-2-one	−0.535	−0.617	−0.200	−0.279	−0.392	−0.207	−0.280	−0.398	−0.992	−1.037	−0.196	−0.372
Hexan-2-one	0.050	−0.131	0.550	0.292	0.104	0.057	0.336	0.092	−0.660	−0.903	0.313	0.057
Heptan-2-one	0.363	0.079	0.929	0.597	0.379	0.240	0.664	0.315	−0.505	−0.862	0.595	0.187
Chloroform	0.089	0.036	0.654	0.494	0.328	0.311	0.552	0.319	−0.639	−0.981	0.480	0.314
Phenylmethanol	−0.233	−0.550	0.195	−0.243	−0.033	−0.197	0.181	−0.216	−0.884	−1.106	0.048	−0.314
Benzaldehyde	−0.044	−0.170	0.418	0.221	0.128	0.152	0.616	0.130	−0.686	−1.058	0.341	0.109
Methyl benzoate	0.338	0.037	0.897	0.488	0.414	0.245	0.765	0.321	−0.561	−0.880	0.608	0.187
Methoxybenzene	0.273	0.066	0.688	0.543	0.376	0.274	0.745	0.349	−0.602	−1.080	0.475	0.232
1-Phenylethanone	0.067	−0.142	0.273	0.247	0.168	0.087	0.474	0.123	−0.673	−0.727	0.395	0.046
1-Phenylpropan-1-one	0.323	0.071	0.709	0.523	0.400	0.278	0.761	0.355	−0.584	−0.690	0.639	0.233
Diphenylmethanone	0.865	0.349	1.364	0.848	0.925	0.543	1.265	0.666	−0.173	−0.578	1.142	0.498
2-Phenylacetonitrile	−0.034	−0.089	0.393	0.284	0.138	0.153	0.410	0.158	−0.709	−0.699	0.360	0.141
Nitrobenzene	0.163	0.026	0.412	0.437	0.343	0.260	0.726	0.313	−0.571	−0.858	0.445	0.092
*p*-Nitrotoluene	0.430	0.194	0.751	0.654	0.552	0.397	0.991	0.495	−0.486	−0.831	0.696	0.350
Fluorobenzene	0.278	0.100	0.981	0.591	0.422	0.310	0.738	0.372	−0.505	−0.868	0.442	0.255
Chlorobenzene	0.564	0.267	1.012	0.817	0.685	0.456	1.071	0.579	−0.477	−0.644	0.823	0.313
Bromobenzene	0.752	0.401	1.339	0.985	0.773	0.605	1.174	0.648	−0.225	−0.778	0.848	0.451
Iodobenzene	0.800	0.419	1.498	1.004	0.909	0.590	1.322	0.760	−0.266	−0.862	0.906	0.533
1-Chloro-4-methylbenzene	0.881	0.452	1.360	1.055	0.947	0.603	1.361	0.778	−0.251	−0.719	0.955	0.552
Benzene	0.231	0.079	0.865	0.485	0.281	0.239	0.493	0.208	−0.613	−1.067	0.382	0.248
Methylbenzene	0.528	0.252	1.195	0.817	0.614	0.429	0.967	0.543	−0.474	−0.925	0.650	0.387
Ethylbenzene	0.802	0.420	1.534	1.028	0.839	0.568	1.229	0.715	−0.331	−0.811	0.885	0.534
Propylbenzene	1.116	0.610	1.894	1.269	1.110	0.724	1.529	0.911	−0.140	−0.699	1.155	0.687
Butylbenzene	1.436	0.804	2.236	1.499	1.379	0.879	1.809	1.111	0.088	−0.603	1.423	0.840
1,4-Dimethylbenzene	0.841	0.431	1.589	1.052	0.881	0.544	1.278	0.739	−0.325	−0.849	1.024	0.523
Biphenyl	1.175	0.601	1.943	1.219	1.226	0.751	1.751	0.958	−0.044	−0.606	1.309	0.730
1,3,5-Trimethylbenzene	1.079	0.631	1.928	1.276	1.138	0.714	1.599	0.940	−0.129	−0.837	1.147	0.657
Bicyclo[4.4.0]deca-1,3,5,7,9-pentene	0.834	0.419	1.560	0.999	0.944	0.597	1.393	0.754	−0.232	−0.624	0.960	0.552
Phenol	−0.290	−0.446	0.186	−0.084	0.181	−0.036	0.238	−0.114	−0.727	−1.191	0.007	−0.165
3-Methylphenol	−0.015	−0.270	0.506	0.113	0.271	0.101	0.505	0.060	−0.632	−1.136	0.218	−0.040
4-Methylphenol	−0.008	−0.270	0.509	0.111	0.287	0.101	0.513	0.065	−0.616	−1.114	0.218	−0.046
2-Methylphenol	0.013	−0.221	0.547	0.173	0.295	0.131	0.561	0.112	−0.621	−1.136	0.238	0.004
4-Ethylphenol	0.268	−0.088	0.833	0.328	0.518	0.251	0.773	0.246	−0.480	−1.058	0.453	0.103
4-Chlorophenol	0.194	−0.153	0.729	0.234	0.525	0.245	0.831	0.229	−0.428	−1.019	0.372	0.054
2-Chlorophenol	0.040	−0.202	0.561	0.182	0.372	0.207	0.620	0.148	−0.614	−1.067	0.274	0.026
3-Chlorophenol	0.216	−0.126	0.743	0.267	0.556	0.277	0.854	0.250	−0.417	−1.049	0.392	0.027
3,5-Dichlorophenol	0.765	0.174	1.391	0.644	0.896	0.586	1.077	0.642	−0.185	−0.903	0.798	0.332
4-Iodophenol	0.422	−0.005	0.995	0.396	0.616	0.364	1.103	0.425	−0.273	−0.966	0.575	0.180
Phenylamine	−0.283	−0.326	0.063	−0.018	−0.096	−0.099	0.121	−0.075	−0.806	−1.125	0.026	0.009
*N,N*-Dimethylaniline	0.480	0.182	1.055	0.727	0.487	0.354	0.918	0.515	−0.448	−1.028	0.675	0.332
1,2-Dimethylbenzene	0.780	0.394	1.509	0.990	0.829	0.525	1.240	0.690	−0.277	−0.888	0.852	0.501
Benzamide	−0.530	−0.742	−0.545	−0.544	−0.301	−0.302	−0.104	−0.550	−0.884	−1.080	−0.164	−0.532
Benzonitrile	0.048	−0.094	0.404	0.283	0.145	0.126	0.396	0.148	−0.486	−0.906	0.264	0.082
2-Methylaniline	−0.087	−0.310	0.364	0.173	0.097	0.031	0.397	0.097	−0.690	−1.100	0.186	0.061
3-Methylaniline	−0.013	−0.186	0.395	0.194	0.121	0.040	0.394	0.093	−0.686	−1.100	0.218	0.069
4-Methylaniline	0.057	−0.186	0.419	0.231	0.131	0.029	0.398	0.090	−0.651	−1.080	0.326	0.048
Furan	−0.219	−0.204	0.327	0.155	−0.012	0.122	0.021	0.232	−0.827	−0.910	0.026	0.023

### LSER Results

**Fig. 2:**
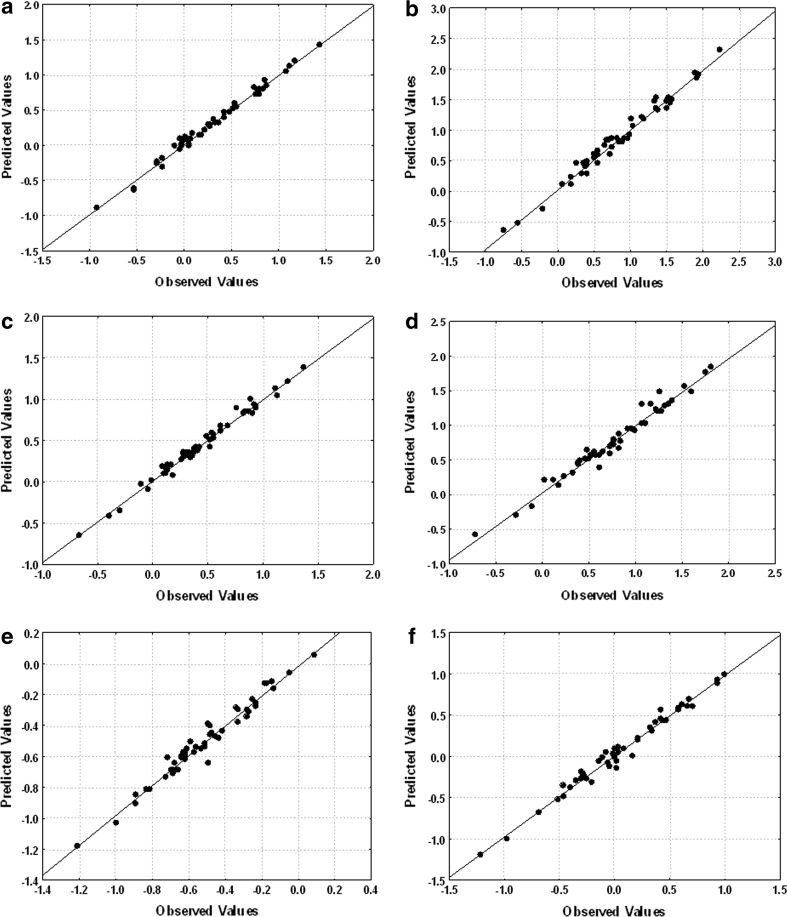
Plots of experimental versus calculated log *k* for MP1: **a** SG-C_18A_, **b** SG-C_18B_, **c** SG-AP, **d** SG-CHOL, **e** SG-P-C10, **f** SG-Ph

**Fig. 3:**
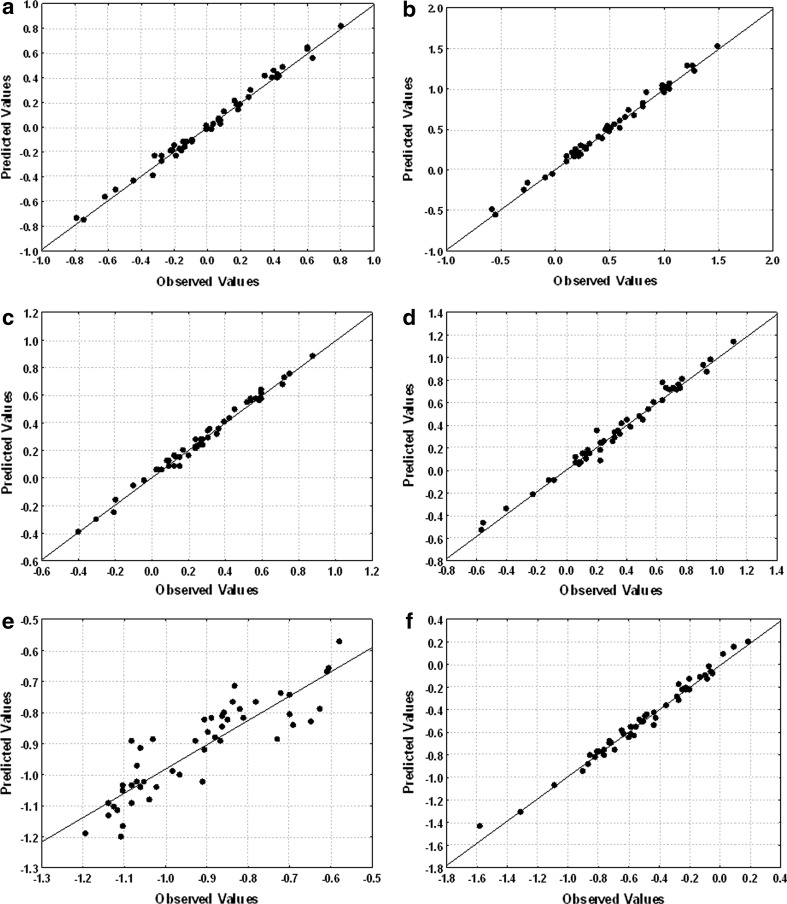
Plots of experimental versus calculated log *k* for MP2: **a** SG-C_18A_, **b** SG-C_18B_, **c** SG-AP, **d** SG-CHOL, **e** SG-P-C10, **f** SG-Ph

Results of the MLR analysis for 50 analytes and six stationary phases are summarized in Table 5. Determination coefficients of the goodness of fit for all the equations are high (0.884–0.995). They are good enough to indicate that LSER method is a suitable approach to identify chemical interactions in HPLC for studied solutes. Figures 2 and 3 present the dependencies of experimentally determined log *k* versus calculated from LSER equations (Table 4) log *k*. The SG-P-C10 gave the poorest fit among all the columns used in the study. This is probably connected with low and close to dead volume *k* values. In case of the rest stationary phases the fits were very good.

**Table 5:** Fitting coefficients according to LSER Eq. (1) for all stationary phases and two mobile phases (MP1 and MP2) used in the study

Stationary phase	*c*	*e*	*s*	*a*	*b*	*v*	*R* ^*2*^	*S*	*F*	*p*
SG-C_18A_ MP1	−0.960	0.150	−0.362	−0.268	−1.763	2.178	0.9890	0.122	793.51	0.00
	±0.046	±0.012	±0.060	±0.035	±0.066	±0.049				
SG-C_18A_ MP2	−0.645	0.050	−0.202	−0.449	−1.413	1.369	0.9900	0.061	878.63	0.00
	±0.032	±0.002	±0.042	±0.025	±0.046	±0.035				
SG-C_18B_ MP1	−0.484	0.192	−0.923	−0.139	−2.102	2.696	0.9785	0.403	402.22	0.00
	±0.083	±0.077	±0.109	±0.064	±0.120	±0.090				
SG-C_18B_ MP2	−0.335	0.082	−0.417	−0.548	−1.719	1.784	0.9913	0.090	1003.71	0.00
	±0.039	±0.036	±0.051	±0.031	±0.057	±0.043				
SG-AP MP1	−0.639	0.106	−0.303	−1.028	−1.690	1.839	0.9852	0.127	586.91	0.00
	±0.046	±0.029	±0.061	±0.036	±0.067	±0.051				
SG-AP MP2	−0.292	0.600	−0.165	−0.208	−1.308	1.088	0.9899	0.038	864.90	0.00
	±0.025	±0.038	±0.034	±0.019	±0.037	±0.028				
SG-CHOL MP1	−0.640	0.243	−0.357	−0.187	−1.912	2.197	0.9701	0.385	585.68	0.00
	±0.081	±0.076	±0.089	±0.063	±0.118	±0.088				
SG-CHOL MP2	−0.388	0.099	−0.241	−0.336	−1.525	1.412	0.9825	0.116	496.20	0.00
	±0.044	±0.041	±0.058	±0.035	±0.064	±0.048				
SG-P-C10 MP1	−1.299	−0.057	0.334	−0.381	−1.093	1.205	0.9654	0.108	645.62	0.00
	±0.043	±0.006	±0.056	±0.033	±0.062	±0.047				
SG-P-C10 MP2	−1.368	−0.183	0.350	−0.409	−0.640	0.576	0.7815	0.302	131.49	0.00
	±0.071	±0.067	±0.034	±0.055	±0.085	±0.078				
SG-Ph MP1	−0.637	1.125	−0.172	−0.357	−1.433	1.842	0.9846	0.120	565.99	0.00
	±0.045	±0.042	±0.059	±0.035	±0.065	±0.049				
SG-Ph MP2	−0.334	0.093	−0.206	−0.389	−1.136	1.088	0.9707	0.126	791.78	0.00
	±0.046	±0.011	±0.061	±0.036	±0.067	±0.051

Coefficients *e, s, a, b, v* in Abraham equation indicate on the preferable interactions of solute with the mobile or stationary phase. If the mobile phase property has a greater value than stationary phase, the coefficient is of negative sign. Opposite situation may be observed when *e, s, a, b, v* coefficient is positive. It proves that stationary phase property exceeds that of the corresponding mobile phase property.

### SG-C_18A_ and SG-C_18B_

Octadecyl stationary phases are the most commonly used among scientists working with HPLC. Properties of this stationary phase are well known, as well as its retention mechanism. Octadecyl packings were also the subject of several LSER studies [3–7]. Application of SG-C_18_ in present investigation had one purpose: we have used it as so-called ‘reference’ material. We have tried to compare other phases with octedecyl one, as it is described in the most complex manner in the literature.

Two octadecyl columns have been used differing in carbon content on modified support surface. The comparison of alkyl chain content on silica surface allowed to observe differences in LSER equation. The trends of *e, s, a, b, v* constants values are very characteristic and typical for octadecyl columns. The *r* and *v* coefficients are the only system properties of positive sign (Table 5). *v* has much higher value in comparison with *e*. Other coefficients are negative, with great *b*. *e, a, s* have low and close to zero numerical values and it proves that this phase acts as a material for immobilizing mobile phase composition (Table 5). Such effect indicates that retention of analyte is not influenced in great extent by excess molar refraction, dipolarity/polarizability and hydrogen-bond acidity.

In case of SG-C_18B_ packing *v* is much higher and *b* much lower in comparison with SG-C_18A_ (Table 5). The nonpolar term *v* reflects favorable solute transfer from the mobile phase to stationary phase, because it is positive and larger than intercept. This proves that interactions between analyte and packing material overcome the energy required for breaking molecular interactions between stationary and mobile phase. Such differences in exoergic dispersive effects lead to positive *v* values. The higher *v* for SG-C_18B_ is also easy to explain in the context of greater carbon load (Table 2).

The large negative *b* coefficient value indicates that bonded phase is a much weaker hydrogen bond donor compared to the mobile phase (Table 5). In case of SG-C_18A_ more residual silanols are available during chromatographic process, since it has low carbon content (Table 2). Moreover, water molecules can interact with residual silanols via hydrogen bonds. Such interaction occurs also with acetonitrile or methanol molecules, consequently their hydrogen bond acidity is reduced.

### SG-AP

Alkylamide stationary phase (Fig. 1) consists of long (twelve carbon atoms) alkyl chains, aminopropyl and amide groups and residual silanols*. s* and *a* coefficients values are higher than in the case of both octadecyl columns (Table 5). More polar character of SG-AP in comparison with SG-C_18A_ and SG-C_18B_ causes greater *s* and *a*.

On the other hand *e* is higher than for SG-C_18A_ and lower than for SG-C_18B_ (Table 5). *e* coefficient refers the ability to interact by electron pairs. SG-AP has one site able to interact by these types of interactions: nitrogen with lone electron pair.

*v* coefficient is lower for SG-AP in comparison with both octadecyl phases (Table 5). This effect is expected, since the polar groups (aminopropyl or amide) favor the incorporation of solvent molecules, which enhances the cohesivity of the stationary phase. Consequently cavity formation becomes more difficult. Moreover, the carbon load on SG-AP is also lower in comparison with octadecyl packings (Table 2). This will have impact on hydrophobic interactions and *v* parameter.

On the other hand *b* coefficient has greater (less negative) values for SG-AP than for SG-C_18A_ and SG-C_18B_ (Table 4). This effect is strictly connected with phenomenon described earlier. It indicate that bonded phase is stronger hydrogen bond donor (compared to the mobile phase components) than both octadecyl columns used in the present investigations. Probably greater amount of water molecules sorbs into the stationary phase surface (aminopropyl or amide groups) relative to the organic solvent molecules. Therefore, the hydrogen bond acidity is greater in case of SG-AP, than for alkyl phases.

### SG-CHOL

Cholesterol stationary phase contains several functional groups bonded to the silica gel surface (Fig. 1). The most important and meaningful is the cholesterol molecule; however, aminopropyl and amide groups, as well as residual silanols are also present (Fig. 1). *e* and *a* values are higher for SG-CHOL in comparison with SG-C_18A,_ SG-C_18B_ and SG-AP (Table 5). Polarizability reflects the favor partition into the stationary phase. The nitrogen atom of aminopropyl group is an active centre, similar as in case of SG-AP. SG-CHOL poses also another possibility to interact by electron pair, since in the structure of cholesterol molecule there is a double bond, with *n*- and *π*-electrons. Therefore, the solute will interact stronger with the stationary phase than with mobile phase solvents. This interaction type influences retention of analytes on SG-CHOL in greater extent in comparison with three earlier described phases.

*s* coefficient on SG-CHOL is similar for SG-C_18A_ and higher for SG-C_18B_ (Table 5). This coefficient is connected with the dipolar interactions and reflects dipolarity/polarizability. Although stationary phase seems to be medium polar, during the chromatographic process it becomes moderately polar, as a consequence of sorption of solvent molecules on stationary phase ligands. Dipolar interactions are greater for SG-AP than for SG-CHOL. On the other hand these interactions are lower for SG-C_18B_ (Table 4). It is connected with the stationary phase structure (Fig. 1). Mobile phase molecules will be preferably sorbed on packing containing more polar groups in its structure. Since cholesterol molecule is large and non polar, SG-AP will be able to interact by dipolar interactions in greater extent in comparison with SG-CHOL.

*v* coefficient for SG-CHOL is lower than for both octadecyl packings, on the other hand *b* is higher (Table 5). The same effect was observed for SG-AP and may be explained by the same reasons. If one would compare *v* and *b* values obtained on SG-CHOL and SG-AP, it appears that second phase is more polar (lower *v*) and hydrogen bond donor interactions are predominant between solute and mobile phase (Table 5). It may be concluded that large cholesterol molecule causes more hydrophobic character of packing surface in comparison with SG-AP; however, SG-CHOL is not as hydrophobic as SG-C_18_. Surprisingly, SG-C_18B_ and SG-CHOL have similar carbon content of modified silica surface. On the other hand SG-CHOL poses also polar aminopropyl groups, which are active centers during chromatographic process (expressed by *e* and *s* coefficients), thus reducing hydrophobic potential of prepared packing material.

### SG-P-C10

SG-P-C10 poses aminopropyl groups and a phosphate group to which alkyl chain was bonded. Therefore, this is another type of HPLC packing with mixed properties, as it is expected from its structure (Fig. 1).

Although SG-P-C10 poses alkyl chain of ten carbons in it structure, apparently the presence of phosphate and amino groups notably increases the polarity. It was confirmed also by the LSER coefficients values (Table 5). Surprisingly, *e* became negative indicating that electron pair interactions play considerable role mainly between mobile phase and solute. Similar situation concerns hydrogen-bond acidity expressed by *a*. Increasing of both properties of solute will negatively influence the log *k* values.

Moreover *s* parameter is low, but positive. Consequently high dipolarity of solute increases its partitioning to the stationary phase. This coefficient is connected with the dipolar interactions, which have the greatest impact on retention for SG-P-C10 among all the stationary phases used in the investigation (Table 5). It is strictly connected with the stationary phase structure and favorable sorption of mobile phase molecules by SG-P-C10 polar groups. Interesting is almost equal carbon load on SG-C_18A_ and SG-P-C10 (Table 2), which may suggest comparable retention. However, on the surface of second packing amino and phosphate groups are also localized (Fig. 1). Therefore, it can be summarized that the differences between these two stationary phases are mainly a consequence of polar groups.

*v* parameter remains positive, like in case of all stationary phases. However, this coefficient is lower than for SG-C_18A_, SG-C_18B_, SG-CHOL, SG-AP, and SG-Ph (Table 5). Consequently hydrophobic interactions of solute with SG-P-C10 are not as strong like in case of other packing materials.

### SG-Ph

SG-Ph poses aryl rings chemically bonded to the silica surface (Fig. 1). Here the π–π interactions are supposed to have the greatest influence. Table 5 presents MLR results for SG-Ph and both mobile phases used under the study. Observed trends in the view of the coefficients sign are similar as for octadecyl, cholesterol or alkylamide phases. Only two parameters are positive: *e* and *v*, while the rest of LSER coefficients have negative values. *v* and *b* are the most significant, like in case of other stationary phases typical for RP HPLC (Table 5). Cavity effect and hydrophobic interactions are the lowest among most of the stationary phases used, it exceeds only *v* values for SG-P-C10. It proves that cavity formation and dispersion interactions of analyte and stationary phase are for SG-Ph not as strong as for SG-C_18_ and SG-CHOL. Such effect is strictly connected with carbon load on support surface (Table 2). The *P*_*C*_ for SG-Ph equals 11.75 %, while in case of SG-C18B and SG-CHOL it is higher and equal to about 17 %. Moreover, *v* coefficient seems to be similar as in case of SG-AP (Table 5), although both phases are different in the structure and nature. Data presented in Table 2 show clearly that both SG-AP and SG-Ph have similar carbon content, therefore *v* is also similar. Hydrogen bond acidity is more meaningful when interactions between mobile phase and solute are considered. It has to be, however, noticed that *b* values are relatively high when this parameter will be compared with other stationary phases (Table 5). Only for SG-P-C10 *b* parameter is higher. Regarding the relatively small positive *e* values it can be concluded that solute interactions via electron pairs (*π*- and *n*-electron pairs) increase partitioning into stationary phase as expected given the nature of stationary phase. As it was summarized in former paragraphs dipolarity-polarizability and hydrogen-bond basicity of solute decrease the retention.

### The Influence of Organic Solvent Type

We have compared also the influence of the type of organic solvent used in mobile phase. Two different solvents were used during the investigations, namely methanol (MP1) and acetonitryl (MP2). These solvents are polar ones (π for methanol equals 0.60 and for acetonitrile 0.75), but they have different hydrogen bonding properties, as well as basicities.

It is well known that changes made in the mobile phase components have significant effect on intermolecular interactions between solute, mobile and stationary phase. Such effect was also observed during present study. It may be distinguished on the basis of results presented in Table 4.

Major differences are seen in the *b* and *v* parameters. First coefficient is higher for MP2 in case of all stationary phases used in the study. This proves that acetonitrile is weaker hydrogen bond donating solvent. Such effect is strictly connected with two phenomenons taking place during the chromatographic process. When methanol is used as a mobile phase component, it sorbs preferentially (in comparison with acetonitrile) into the stationary phase ligands. Therefore, methanol takes part in imparting hydrogen bond donating ability to stationary phase. Acetonitrile also modify stationary phase, nevertheless it does not have significant hydrogen bond donating strength. However, there is also another effect, which may influence the value of *b* coefficient. The ability to hydrogen bond donation may arise from the presence of residual silanols groups. These groups adsorb water or water associated with organic solvent, and consequently take part in hydrogen bond interactions.

As it was already stated *v* parameter is noticeably lower for MP2. This effect is a consequence of greater elution strength of acetonitrile in comparison to methanol. Therefore, the strength of hydrophobic interactions is reduced when this solvent is used as a mobile phase component.

*e* coefficient is also lower for MP2 than for MP1 in case of all columns used in the investigations. As it was summarized earlier this coefficient reflects the ability to interact by electron pair (by *n*- or *π*-electrons). It appears that such a possibility is greater for methanol. There is just one lone electron pair in the structure of acetonitrile (nitrogen atom) and two of them in methanol (oxygen atom).

On the other hand *s* parameter is higher for MP2 in comparison with MP1. However, this situation concerns only SG-C_18A_, SG-C_18B_, SG-AP, and SG-CHOL. In case of SG-Ph, SG-P-C10 *s* is almost similar for both mobile phases, therefore dipole interactions and induction effects are analogous for these packing materials in MP1 and MP2. Situation with *a* coefficient seems to be related. SG-C_18A_, SG-C_18B_, SG-AP, and SG-CHOL have higher *a* parameter for MP1, while in the case of the rest of stationary phases this value is similar. It means that usage of any of these mobile phases will cause analogous interactions by hydrogen bond acceptor.

## Conclusions

Interactions determining retention on specific stationary phase were successfully studied with the use of LSER model. Stationary phases used in the investigations are structurally very different. However, it has to be pointed out that for several of them significant differences in interactions were not observed. It concerns SG-C_18A_, SG-C_18B_, SG-AP, SG-CHOL and SG-Ph. The *v* coefficient is always the largest and of positive sign, thus exerting the greatest influence on retention (also for polar SG-P-C10). The only other coefficient that can increase the retention for SG-C_18A_, SG-C_18B_, SG-AP, SG-CHOL and SG-Ph is *r* parameter. Characteristic to these phases is that the acidicity is insignificant in contrast to basicity, which is the main hydrophilic term. *b* value is always of negative sign. It may be concluded that polar interactions have negative effects to retention.

Different situation occurs for SG-P-C10. It has relatively low (when compared with other stationary phases used in the study) *v* and high *b*, although it remains with negative sign. The structure of SG-P-C10 poses several polar groups, therefore polar interactions play more important role than in case of SG-C_18A_, SG-C_18B_, SG-AP, SG-CHOL and SG-Ph. High dipolarity of solute increases its partitioning to stationary phase.

Major differences, concerning the type of organic modifier in mobile phase, are seen in the *b* and *v* parameters. First coefficient is higher for MP2 in case of all stationary phases used in the study, while *v* is lower for MP2.
